# A Channel Allocation Method Considering the Asymmetry of Available Channels for Centralized Underwater Cognitive Acoustic Networks

**DOI:** 10.3390/s23063320

**Published:** 2023-03-21

**Authors:** Changho Yun

**Affiliations:** Korea Research Institute of Ships & Ocean Engineering (KRISO), Daejeon 34103, Republic of Korea; sgn0178@kriso.re.kr; Tel.: +82-42-866-3834

**Keywords:** channel, cognitive user, channel allocation, queueing model, resource allocation, underwater acoustic frequency band, Underwater Cognitive Acoustic Network

## Abstract

Due to the unpredictable presence of Non-Cognitive Users (NCUs) in the time and frequency domains, the number of available channels (i.e., channels where no NCUs exist) and corresponding channel indices per Cognitive User (CU) may differ. In this paper, we propose a heuristic channel allocation method referred to as Enhanced Multi-Round Resource Allocation (EMRRA), which employs the asymmetry of available channels in existing MRRA to randomly allocate a CU to a channel in each round. EMRRA is designed to enhance the overall spectral efficiency and fairness of channel allocation. To do this, the available channel with the lowest redundancy is primarily selected upon allocating a channel to a CU. In addition, when there are multiple CUs with the same allocation priority, the CU with the smallest number of available channels is chosen. We execute extensive simulations in order to investigate the effect of the asymmetry of available channels on CUs and compare the performance of EMRRA to that of MRRA. As a result, in addition to the asymmetry of available channels, it is confirmed that most of the channels are simultaneously available to multiple CUs. Furthermore, EMRRA outperforms MRRA in terms of the channel allocation rate, fairness, and drop rate and has a slightly higher collision rate. In particular, EMRRA can remarkably reduce the drop rate compared to MRRA.

## 1. Introduction

Underwater acoustic communication can provide longer-range and more reliable data transmission than other methods, such as optical and radio-frequency communication [1,2]. However, collisions with various interferers, such as sonar devices, ship noise, or underwater mammals, are still a major issue that affects the efficiency of a narrow bandwidth in underwater acoustic communication [3,4]. The demand for underwater acoustic communication is also growing due to the expanding use of underwater applications, including monitoring ecosystems and disasters, developing ocean resources, detecting threats, and building underwater structures [5,6]. In addition, there is a need to develop eco-friendly underwater acoustic communication that reduces harm to underwater mammals such as dolphins [7,8].

Cognitive Radio Networks (CRNs) aim to improve temporal–spatial spectral efficiency by allowing non-licensed users to access spectrum holes [9,10]. An Underwater Cognitive Acoustic Network (UCAN) is a technology that enables a Cognitive User (CU) to dynamically determine its acoustic frequencies based on channel conditions, quality of service (QoS), and the presence of interferences [11,12]. UCAN can increase the utilization of acoustic frequencies and reduce harm to the underwater ecosystem [13].

The development of UCAN technologies is challenging because of the following differences from CRNs, as specified in [14]:

- While there are well-known channel models in CRNs, predicting the channel model in UCANs is difficult due to the presence of unpredictable multi-paths and diverse noises;
- CRNs have a channel plan that defines the center frequency, channel index, and bandwidth, while in UCANs, the acoustic frequency band is an open spectrum where overlapping use of frequencies is inevitable;
- In CRNs, primary and secondary users are clearly differentiated by the license policy, while in UCANs, there are unpredictable and uncontrollable interferers due to the lack of a standardized channel plan;
- In CRNs, any signal can be decoded due to the standardized signaling format, while in UCANs, most signals from neighboring interferers are uninterpretable.

Since the introduction of the need for UCANs in [13], a range of UCAN technologies have been studied. In the literature, the majority of works focus on proposing efficient resource allocation methods that assign a CU to network resources (e.g., acoustic frequency, data rate, and transmission power). In [14], previous UCAN studies are categorized into three groups: single resource allocation, joint resource allocation, and other than resource allocation. We have included additional existing works and have summarized their characteristics in Table 1.

In particular, Ref. [14] defined the concept of interferers (i.e., NCUs) and fragmented time domain units, and derived important considerations of spectrum sharing for centralized UCANs. These considerations can be used to develop other protocols, such as spectrum mobility or channel access. Unlike prior optimization-based approaches, Ref. [14] also proposed a heuristic spectrum sharing protocol, which consists of how to set the allocation order of CUs and the way in which to assign channels to them. Simulation results showed that the combination of a low channel allocation rate (CAR)-based allocation priority and multi-round resource allocation (MRRA) outperformed other pairs in terms of CAR and fairness. The low-CAR-based allocation priority gives a CU’s channel allocation priority in ascending order of CAR, and MRRA involves a central entity allocating a channel to a CU in each round until no more channels are needed or there is a lack of channels available.

Let us define an available channel as one where there are no NCUs present after channel sensing. Due to the random occurrence of NCUs in the time and frequency domains, the number of available channels per frame and the corresponding channel indices for a CU can vary. This is defined as the asymmetry of available channels among CUs. In MRRA, a central entity randomly selects one channel from a CU’s available channels and allocates it, ignoring this asymmetry. However, this asymmetry can impact the efficient and fair allocation of limited underwater acoustic channels among CUs. For instance, if the central entity controls the number of required channels based on the number of available channels, it can help the CU to prevent data from being dropped in the queue. Moreover, when there are two CUs with the same allocation priority, allocating to the CU with fewer available channels can improve the fairness of spectrum sharing. To the best of our knowledge, no existing resource allocation method for UCANs takes into account the asymmetry of available channels, as shown in Table 1.

This paper primarily investigates the impact of the asymmetry of available channels on UCANs. A new channel allocation method, Enhanced MRRA (EMRRA), is proposed to reflect the asymmetry of available channels among CUs in MRRA. The following summarizes the contents of the paper:

- By varying the number of NCUs, the number of channels, and the number of CUs in simulations, we analyze the relationship between the asymmetry of available channels and simulation conditions. In addition, the number of available channels and the redundancy of available channels among CUs are investigated with respect to simulation conditions.
- EMRRA is designed based on two ideas: first, among CUs with the same allocation priority, a CU with the smallest number of available channels is selected preferentially to increase the probability of channel allocation and enhance fairness. Second, when a channel is allocated to one CU, the available channel with the lowest redundancy with other CUs is determined to improve overall spectrum utilization.
- Simulations based on a queueing model are performed. By employing a low-CAR allocation priority, the performance of EMRRA and MRRA is analyzed in terms of CAR, fairness, drop rate (DR), and collision rate (CR). The results show that EMRRA outperforms MRRA in terms of CAR, fairness, and DR. In particular, EMRRA results in a remarkable DR performance improvement. EMRRA may result in a slightly higher CR than MRRA due to the allocation of more channels, which increases the probability of collisions.

The contributions of this paper are summarized as follows:

- This study identifies the asymmetry of available channels in UCANs and evaluates its impact through simulations. The concept of the asymmetry of available channels can be useful in the design of future protocols for UCANs.
- We propose a new channel allocation method, EMRRA, which takes into account the asymmetry of available channels, which has not been considered in previous methods. The proposed method is evaluated through simulations and is shown to be superior in terms of channel allocation rate, fairness, and drop rate.

The structure of the paper is as follows. Section 2 provides a system model and an explanation of the concept of the asymmetry of available channels. Section 3 presents the details of the proposed EMRRA method. Section 4 describes simulations to investigate the asymmetry of available channels and evaluate the performance of EMRRA and MRRA. Finally, the paper concludes in Section 5.

## 2. System Model and Asymmetry of Available Channels

### 2.1. System Model

We consider a three-dimensional centralized UCAN, as the same as in [14]. The network is composed of one central entity and multiple CUs. The central entity can be an underwater base station, sink node, or cluster head, and CUs can be sensor nodes, divers, or autonomous underwater vehicles. To carry out cognitive communication, the central entity and CUs have the ability to sense the overall underwater acoustic frequency band and choose a desired acoustic frequency.

**Figure 1:**
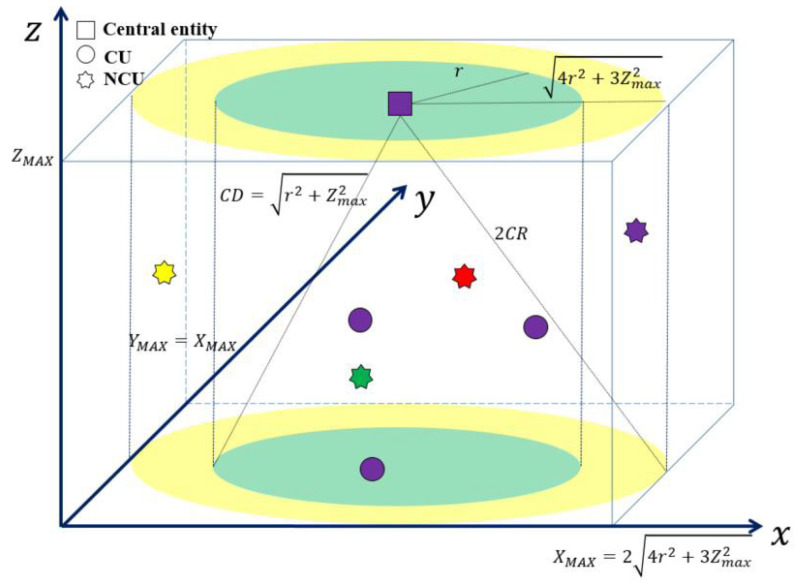
The topology of a centralized UCAN.

The central entity is positioned at the center of the water surface in the three-dimensional centralized topology, as shown in Figure 1. CUs are located in a cylinder with a radius of r and height of $Z_{max}$. The central entity and CUs are situated in a location where one-hop communication is possible, and the communication distance (CD) for all CUs is defined as $\sqrt{r^{2}+Z_{max}^{2}}$. The location of a CU and central entity is represented in x-y-z coordinates and the maximum values of the x and y axes are expressed as r and $Z_{max}$, as shown in Figure 1. The *x* and *y* coordinates of a CU are randomly set in the range of $\left[\sqrt{{4r}^{2}+{3Z}_{max}^{2}}-r,\sqrt{{4r}^{2}+{3Z}_{max}^{2}}+r\right]$, and the *z* coordinate of a CU is arbitrarily determined in the range of $\left[0,Z_{MAX}\right]$.

Only NCUs within an area with twice the CD can be detected, so we consider NCUs located in a cylinder with a radius of $\sqrt{4r^{2}+3Z_{max}^{2}}$ and a height of $Z_{max}$. In this area, NCUs randomly occur in both the time and frequency domains. In Figure 1, the star-shaped symbols depicted in different colors indicate that NCUs occur in different frequency bands. The x and y coordinates of an NCU are randomly set in the range of $\left[0,2\sqrt{{4r}^{2}+{3Z}_{max}^{2}}\right]$, and the z coordinate of an NCU is randomly set in the range of $\left[0,Z_{MAX}\right]$.

**Figure 2:**
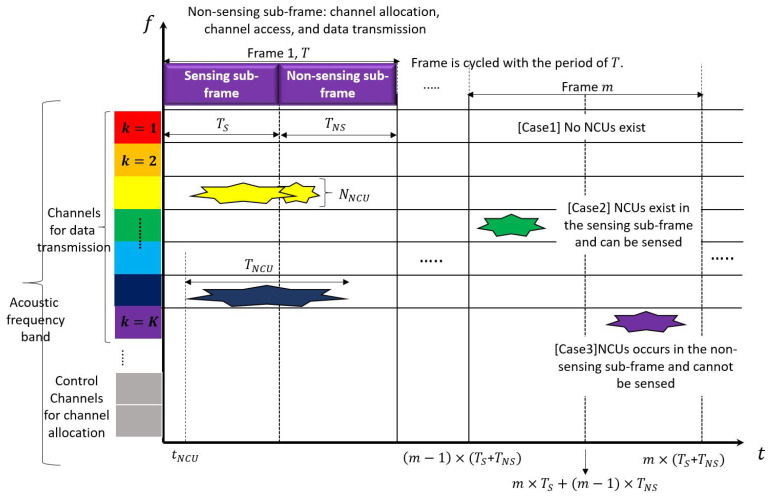
An illustration of the fragmentation in both time and frequency domains.

Figure 2 illustrates the fragmentation in both the time and frequency domains for UCANs. The *x* and *y* axes depict the time and frequency domains, respectively. Each domain is segmented to check the status of NCUs, distinguish the sensing time from the non-sensing time, and assign adequate channels to a CU. The fragmentation of each domain is described as follows:

- Cognitive communication in a UCAN requires segmenting the underwater acoustic frequency band into multiple channels, unlike existing underwater acoustic communication that uses a fixed frequency. Previous works have simply assumed that the underwater acoustic frequency band consists of multiple channels, but a standardized frequency system, such as the one proposed in [3], is necessary for cognitive communication to detect NCUs and prevent them from using available spectrum holes. In this paper, the underwater acoustic frequency band is divided into multiple control channels and multiple channels (as illustrated on the y axis of Figure 2). The control channel is used to transmit CU sensing information to the central entity, or for the central entity to allocate channels to the CU. A channel is utilized for communication between CUs and between CUs and the central entity.
- In addition, it is challenging to predict the status of NCUs, including the duration of their occurrence, time of occurrence, and the number of NCUs on a channel. Thus, repeated sensing is required for underwater cognitive communication to respond to the random activities of NCUs. When adopting the “sensing first, data transmission last” approach of cognitive communication, the sensing and data transmission routine must be performed periodically. To accomplish this, fragmentation of the time domain is also necessary. As is the case in traditional TDMA, the time domain is segmented into multiple frames, each composed of sensing and non-sensing sub-frames, as shown in Figure 2. The sensing sub-frame is the time for CUs to purely sense NCU activities, with a length defined as *T_s_*. The non-sensing sub-frame, with a length defined as $T_{NS}$, is the time for CUs to transmit sensing information to the central entity, for the central entity to assign channels to CUs, or for CUs to transmit and receive data via the channels. The length of a frame, T, is defined as $T=(1+\alpha )\times T_{NS}$, where α is the ratio of the sensing sub-frame length to the non-sensing sub-frame length. As depicted in the lower-right part of Figure 2, the start time point, the mid time point, and the end time point of the $m_{th}$ frame are individually defined in terms of m, $T_{S}$, and $T_{NS}$.

The activity of an NCU is modeled in terms of the number of occurring NCUs ($N_{NCU}$), the occurrence time of each NCU ($t_{NCU}$), and the occurrence time duration of each NCU ($T_{NCU}$), as described in [14]. The variables $t_{NCU}$ and $T_{NCU}$ are modeled to have a uniform distribution in the range of $\left[\left(m-1\right)\times T,m\times T\right]$ and $\left[1,T_{MAX}\right]$, respectively, where $T_{MAX}$ is the maximum occurrence time duration. $N_{NCU}$ is modeled to have a Poisson distribution with an average of $\lambda_{NCU}$. As seen in Figure 2, due to the division of the time and frequency domains, three cases can occur at a specific time and on a specific channel: (1) no NCUs exist in a frame; (2) NCUs exist in the sensing sub-frame and can be sensed; (3) NCUs occur in the non-sensing sub-frame and cannot be sensed.

**Figure 3:**
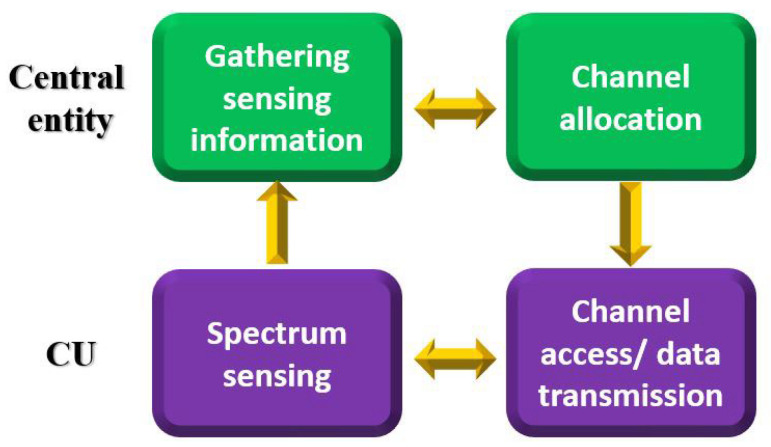
The channel sharing process of a UCAN.

The channel sharing of our target UCAN essentially involves four processes, as illustrated in Figure 3. They are explained as follows:

- A CU operates in two modes, namely the transmission and reception modes. The CU is mostly in the reception mode, except for data transmission, in order to sense a spectrum.
- Spectrum sensing is the process wherein CUs sense all channels in order to check the status of NCUs. Channel state information is exemplified as the signal strength, signal duration, and the CU’s QoS information (e.g., usage time, data rate), and it is used when a central entity makes allocation decisions.
- Gathering sensing information is the process wherein CUs transmit their sensing information to a central entity through a control channel to obtain channels for data transmission. A CU can access the control channel using a predetermined channel access method such as TDMA-based round-robin or random access. After obtaining sensing information from all CUs, the central entity processes the information to assign channels to CUs.
- Channel allocation is the process wherein the central entity determines and allocates appropriate channels to CUs based on the information received from CUs on the control channel. Channel decisions and assignments are made to improve the target performance based on the given channel allocation method. The central entity notifies each CU of the channel allocation results through the control channel.
- Channel access and data transmission is the process wherein a CU transmits and receives data between the central entity and the CU on an allocated channel on the basis of a given channel access method. The central entity continuously monitors channel use in real time and adjusts channel allocation as needed (e.g., the communication failure on the allocated channel due to noise or movement of a CU).
- In cases where processes are proceeding in parallel, a CU continuously updates its channel state information in reception mode when it is not transmitting, and then it periodically transmits channel state information.

### 2.2. The Asymmetry of Available Channels

It is difficult to accurately predict the location of artificial and natural interferences (NCUs) in water. Additionally, decoding the acoustic signals transmitted by NCUs is challenging, due to the lack of a standardized message format. As a result, a channel should be considered as an ON–OFF process to ensure successful data transmission and avoid collisions with NCUs [20,28]. Hence, any channel occupied by an NCU should be deemed as “unavailable” for cognitive communication. This approach is similar to the interweaved channel sharing for CRNs [32,33].

As explained in Section 2.1, the activity of NCUs in both the time and frequency domains is random, making it difficult to predict the exact time and channel of their occurrence, as well as their duration. From the point of view of a CU, both the number of NCUs and available channels experienced by the CU can vary depending on its location. Accordingly, the random activities of NCUs and the different locations of CUs result in all CUs experiencing inequivalent available channels; the number of available channels and corresponding channel indices of CUs are different. This difference in available channels and channel indices for different CUs is referred to as the “asymmetry of available channels”.

An available channel can be classified into two categories based on the number of CUs that consider the channel to be “available”: a non-overlapping available channel (NAC) and an overlapping available channel (OAC). The former is a channel that can be assigned to one CU exclusively, while the latter is a channel that is available to multiple CUs at the same time and requires an appropriate channel allocation strategy.

**Figure 4:**
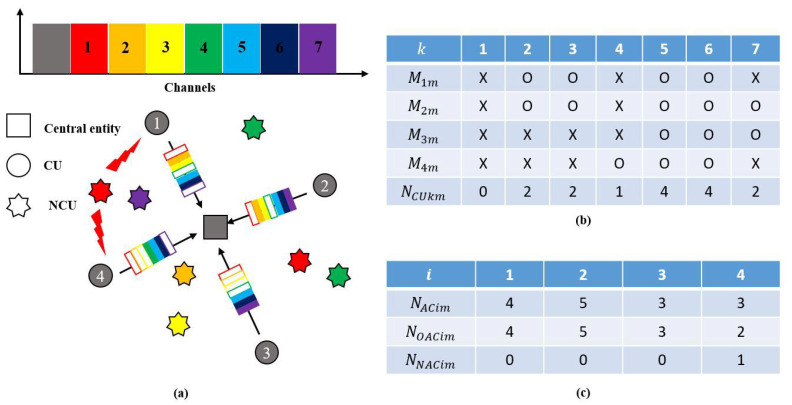
An example of the asymmetry of available channels in a centralized UCAN: (**a**) network configurations and channel description; (**b**) the results of $M_{im}$ and $N_{CUkmx}$; (**c**) the results of $N_{ACim}$, $N_{NACim}$, and $N_{OACim}$.

Figure 4 provides an example of the channel availability of CUs. To explain this example, corresponding parameters are defined in Table 2. The example in Figure 4a shows a centralized UCAN system with four CUs sharing seven channels. As seen in Figure 4a, CU 1 can detect three NCUs at channels 1, 4, and 7. The NCU at channel 4 is detected by both CUs 1 and 2, and the NCUs at channels 1 and 7 are detected by CUs 1 and 4. Based on the example in Figure 4a,b shows the results of $M_{im}$ and $N_{CUkm}$, and Figure 4c shows those of $N_{NACim},N_{OACim},$ and $N_{ACim}$.

Figure 4b illustrates that the values of $M_{im}$ for the four CUs are distinct, and the values of $N_{CUkm}$ for the seven channels are also unequal. Figure 4c shows that the values of $N_{ACim},\;N_{NACim}$, and $N_{OACim}$ for the four CUs are different from each other. This example highlights that the available channels experienced by a CU are different when NCUs are present in different channels around the CU. Furthermore, it can be seen that even with slight variations in the location of NCUs, the number of NCUs, and the channels where NCUs occur, the number of available channels and corresponding channel indices that CUs experience are not consistent and vary among them. The characteristics of the available channel asymmetry will be further explored through simulations in Section 4.

## 3. Enhanced Multi-Round Resource Allocation

In Section 3, EMRRA is described, including the definition of parameters, the queuing model, the method for allocating non-overlapping available channels, and the method for allocating overlapping available channels.

### 3.1. Queueing Model

**Figure 5:**
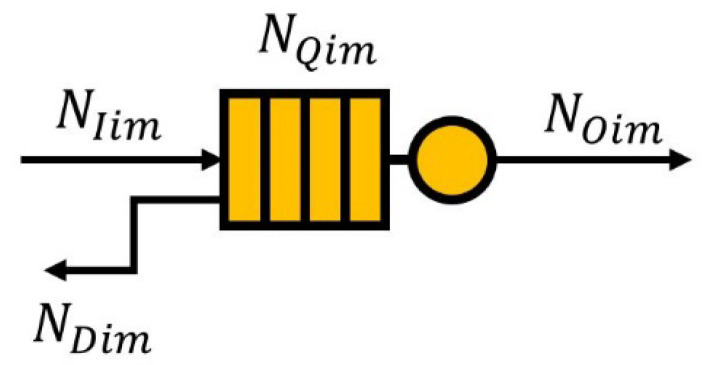
A queueing model of a CU.

In this paper, we define a queuing model for channel allocation purposes, as shown in Figure 5. In other words, the input traffic, output traffic, buffered traffic, and dropped traffic of a queue are expressed in terms of the number of channels; the amount of each type of traffic is associated with the corresponding number of channels.

The parameters of the queueing model are defined as follows:

- $N_{Iim}$ is the number of channels corresponding to the input traffic of queue for CU i at the $m_{th}$ frame.
- $N_{Oim}$ is the number of channels corresponding to the output traffic of queue for CU i at the $m_{th}$ frame. Depending on the sensing results, CU i may not receive any channels if it senses no available channels, or it can be allocated as many as channels as requested.
- $N_{Qim}$ is the number of channels corresponding to the buffered traffic of queue for CU i at the end of the $m_{th}$ frame. $N_{Qim}$ is limited to $N_{Qmax}$ (the maximum queue length), and it is expressed as $N_{Qim}=\sum_{l=1}^{m}\left[N_{Iil}-N_{Oil}\right]$.
- $N_{Dim}$ is the number of channels corresponding to the dropped traffic of queue for CU i at the $m_{th}$ frame. Temporary buffered traffic, $N_{TQim}$, before channel allocation for CU i at the $m_{th}$ frame is defined as $N_{TQim}$ ($=N_{Qi(m-1)}+N_{Iim}$). Traffic can be dropped if $N_{TQim}$ exceeds $N_{Qmax}$, and $N_{Dim}$ is defined as $N_{Dim}=\left\{\begin{array}{c} 0,N_{TQim}>N_{Qmax}\\ N_{TQim}-N_{Qmax},\;otherwise \end{array}\right.$.

The queueing model is described as follows:

- Bulk arrival. $N_{Iim}$ data arrive in batches, and the amount of data in each batch follows a uniform distribution. Moreover, the arrival time of each batch is periodic, implying that each batch arrives at fixed time intervals (T).
- Bulk departure. $N_{Oim}$ data are served in batches. The amount of data served in each batch follows a random distribution because the channel allocation result is random due to the random occurrence of NCUs. Once the service is completed, the entire batch leaves the system. In addition, the departure time of each batch is also periodic, meaning that each batch leaves the system at fixed time intervals (T).
- There is only one server to serve the data.
- The queue model is executed based on the First-In-First-Out (FIFO) principle.
- Finite queue length. If there are data beyond the queue length, this part of the incoming data will be lost.

In an ideal scenario, when a CU is allocated as many channels as $N_{TQim}$ in a given frame, all the traffic in the queue can be processed. However, as the number of CUs increases, the number of allocated channels per CU may decrease, leading to more traffic being stacked in the queue. Additionally, if a CU experiences several NCUs over an extended period, the number of available channels will continue to decrease, resulting in a decrease in the number of allocated channels. This can cause delay and the throughput performance of the CU to degrade as the amount of buffered traffic in the queue grows.

Accordingly, the number of requested channels for CU i at the $m_{th}$ frame to a central entity, denoted as $N_{Rim}$, should be determined by applying both the queuing model and the number of available channels. It is designed so that CU i cannot request as many channels as the number of available channels (i.e., $N_{ACim}$) or the length of the queue before channel allocation (i.e., $N_{TQim}$) at the $m_{th}$ frame. In addition, a CU has no need to request a channel if it either has no available channel or has no traffic to be processed in its queue. Taking these aspects into account, $N_{Rim}$ is defined as $N_{Rim}=\left\{\begin{array}{c} 0,\;N_{TQim}=0\;or\;N_{ACim}=0\\ \min\;(N_{TQim},N_{ACim}),\;otherwise \end{array}\right.$.

### 3.2. Channel Allocation

**Figure 6:**
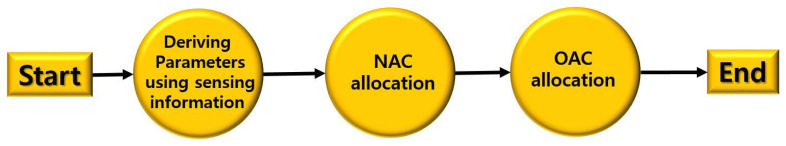
The procedures of EMRRA.

As the activity of NCUs changes with each frame, channels are assigned to CUs adaptively by using sensing information collected in each frame. As explained earlier, the available channels of CUs are divided into overlapping and non-overlapping available channels; the former is proprietary to a CU, but the latter is available to multiple CUs simultaneously. Accordingly, the non-overlapping available channels exclusive to a specific CU are allocated to the CU at first. After this, the overlapping available channels are assigned to CUs using a given allocation method. To do this, EMRRA is composed of channel allocation information analysis, non-overlapping available channel allocation, and overlapping available channel allocation, as depicted in Figure 6. Deriving parameters using sensing information is a procedure of extracting the significant information needed for channel allocation, using sensing information received from all CUs. NAC allocation is a procedure of assigning non-overlapping available channels to corresponding CUs. OAC allocation is a procedure of allocating overlapped available channels to CUs based on the channel allocation rules. These three procedures are sequentially executed, and the details of each procedure are described in Section 3.2.1, Section 3.2.2 and Section 3.2.3, respectively.

Additionally, three parameters are defined as follows:

- $N_{Cim}$ is the number of allocated channels experiencing a collision with NCUs for CU i at the $m_{th}$ ($N_{Cim}\leq N_{Oim}$);
- $S_{1m}$ is the set of CUs of $N_{ACim}>0$ and $N_{Rim}$ > 0 at the $m_{th}$ frame before channel allocation;
- $S_{2m}$ is the set of CUs of $N_{ACim}>0$ and $N_{Rim}$ > 0 at the $m_{th}$ frame during the overlapping available channel allocation.

#### 3.2.1. Channel Allocation Information Analysis

At the $m_{th}$ frame, a CU checks its sensing information (i.e., the existence of NCUs on each channel) after sensing. The CU stores the channel indices where NCUs did not occur in $M_{im}$. Then, the CU transmits $M_{im}$ information together with the current queue length (i.e., $N_{TQim}$) to a central entity. Then, the central entity derives the number of available channels and corresponding channel indices for each CU based on the sensing information received from all CUs, and checks the redundancy of each available channel among CUs. This process determines the $N_{ACim},N_{NACim},$ and $N_{OACim}$ information of all CUs and $N_{CUkm}$ for each channel. The derived channel information is tabulated and updated periodically, as shown in Figure 4b,c.

Using the derived channel information, CUs eligible to receive channels are buffered in $S_{1m}$ using the following steps:

- If either the $N_{ACim}$ or $N_{TQim}$ of a CU is zero, the CU cannot receive any channel in that frame and is excluded from channel allocation candidates;
- As non-overlapping available channels are primarily assigned, the indices of CUs that meet the condition of $N_{NACim}>0$ and $N_{Rim}>0$ are buffered in $S_{1m}$.

#### 3.2.2. Non-Overlapping Available Channel Allocation

**Figure 7:**
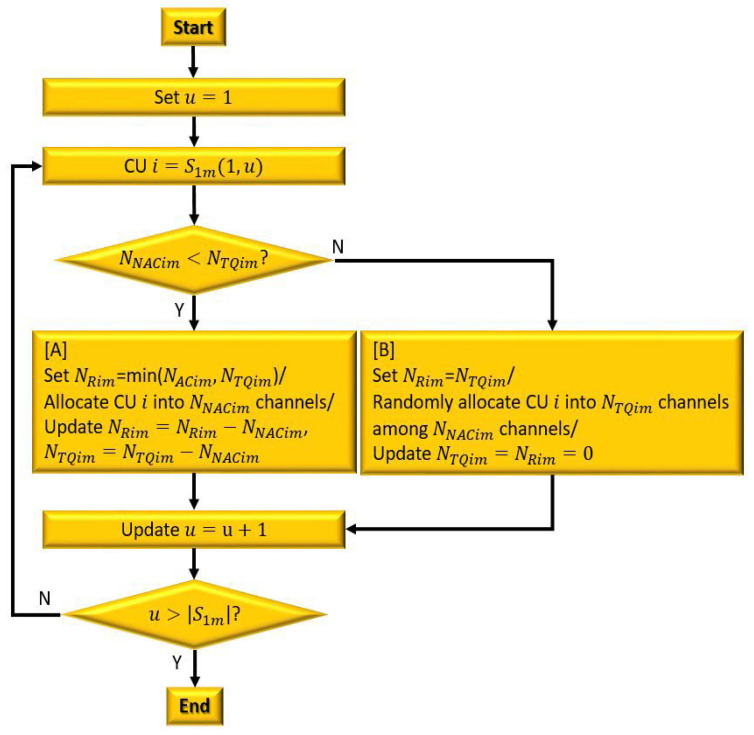
A flow chart of NAC allocation.

The non-overlapping available channel allocation is exclusively available to a specific CU, allowing the CU to allocate as many non-overlapping channels as it requests without restriction. The non-overlapping available channels are assigned to the CUs in $S_{1m}$ in ascending order of CU indices, as shown in Figure 7. If $N_{TQim}\leq N_{NACim}$ (as shown in box [A] in Figure 7), CU i can request $N_{TQim}$ channels (i.e., $N_{Rim}=N_{TQim}$). In this case, as many channels as required can be assigned from the non-overlapping available channels, so that $N_{Rim}$ channels are randomly assigned from $N_{NACim}$ non-overlapping available channels. The corresponding information, including $N_{TQim}$, $N_{Qim}$, and $N_{Rim}$, is also updated to zero.

In the case where $N_{TQim}>N_{NACim}$ (as shown in box [B] in Figure 7), CU i sets $N_{Rim}$ to $\min\;(N_{TQim},N_{ACim})$. Then, it requests $N_{Rim}$ channels from the central entity. In this case, $N_{NACim}$ non-overlapping available channels are assigned first, as in the case of $N_{TQim}\leq N_{NACim}$. The allocation of the remaining $\left({N_{Rim}-N}_{NACim}\right)$ channels is attempted in the next stage (the overlapping available channel allocation). All $N_{NACim}$ channels are assigned to CU i, and both $N_{TQim}$ and $N_{Rim}$ are updated to $N_{TQim}-N_{NACim}$ and $N_{Rim}-N_{NACim}$, respectively. After the non-overlapping available channel allocation is completed, any CU with $N_{Rim}>0$ and $N_{OACim}>0$ is added to $S_{2m}$ to proceed to the next stage.

#### 3.2.3. Overlapping Available Channel Allocation

In the overlapping available channel allocation, multiple CUs can access the same available channel, and the allocation of this channel affects the number of overlapping available channels feasible for the related CUs. For instance, if one CU is assigned a particular overlapping available channel, the other CUs that can access the same channel cannot be assigned it. Thus, a suitable allocation criterion is required for assigning an overlapping available channel.

**Figure 8:**
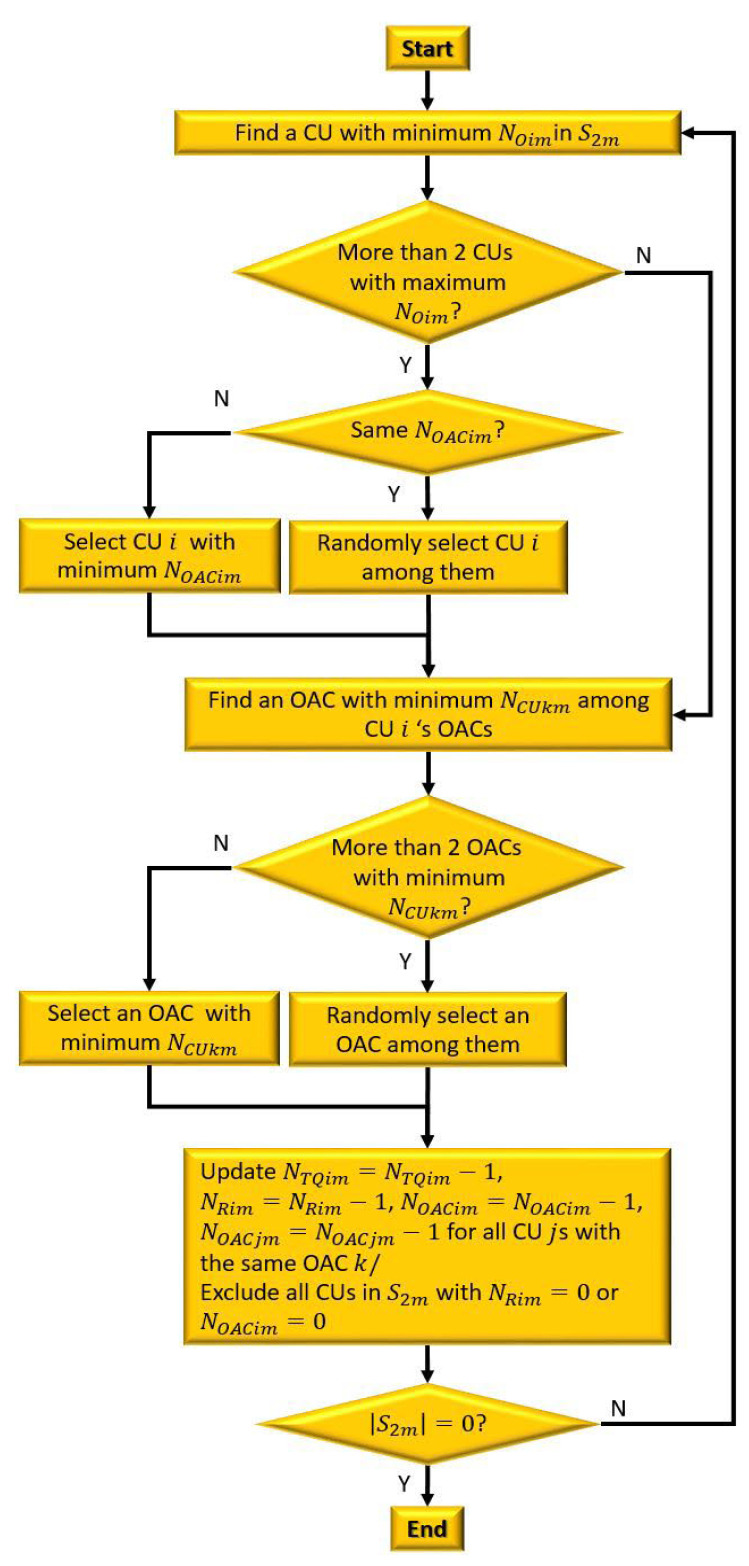
A flow chart of OAC allocation.

An overall process of OAC allocation is illustrated in Figure 8. The allocation of overlapping available channels is executed via the following six steps:

1. In this paper, a low-CAR-based allocation priority that allocates a channel to a CU with the lowest CAR first is applied, as in [14]. Since CAR is defined as the number of currently allocated channels among all channels (i.e., $N_{Oim}/K$), the CU with the lowest $N_{Oim}$ value in $S_{2m}$ is selected without loss of generality.
2. If there are multiple CUs with the same minimum $N_{Oim}$ value, the one with the minimum $N_{OACim}$ value is selected. This is because a CU with a large $N_{OACim}$ value has more overlapping available channels to be assigned, even if it is not allocated this overlapping available channel. Thus, giving higher priority to a CU with the lowest $N_{OACim}$ value can improve the overall channel allocation rate.
3. If there are at least two CUs with the same minimum values of $N_{Oim}$ and $N_{OACim}$ at the same time, one of them is selected randomly.
4. When a central entity allocates a channel to the selected CU i, it chooses the available channel with the minimum $N_{CUkm}$ value. This is because an available channel with a large $N_{CUkm}$ value can be highly probable to be assigned than that with a small $N_{CUkm}$ value. Moreover, this is also intended to boost the overall channel allocation rate of a UCAN. If there are multiple overlapping available channels with the same minimum $N_{CUkm}$ value, one of them is determined in a random fashion.
5. After determining both a CU and a channel to be allocated, the central entity updates each parameter as $N_{TQim}=N_{TQim}-1$, $N_{OACim}=N_{OACim}-1$, and $N_{Rim}=N_{Rim}-1$. The $N_{OACjm}$ and $N_{ACjm}$ of the other CU j that senses the same available channel as CU i are also updated (i.e., $N_{OACjm}=N_{OACjm}-1$ and $N_{ACjm}=N_{ACjm}-1$). After updating, any CU with $N_{Rim}=0$ or $N_{OACim}=0$ is excluded from $S_{2m}$.
6. Repeat the channel allocation process until $S_{2m}$ is empty.

## 4. Performance Analysis

In this section, the features of the asymmetry of available channels are analyzed via simulations. In addition, the performance of EMRRA and MRRA is compared in terms of the channel allocation rate, fairness, drop rate, and collision rate through extensive simulations.

### 4.1. Analyis of Asymmetry of Available Channels

#### 4.1.1. Performance Metrics and Simulation Conditions

To study the asymmetry of available channels, the following performance metrics are considered:

- $\delta_{AC}$ is the average ratio of the number of available channels to that of channels. It represents the average percentage of available channels across all channels. The average number of available channels for all CUs ($N_{AC})$ is defined as $N_{AC}=\frac{1}{N_{SIM}}\sum_{m=1}^{N_{SIM}}\frac{1}{N_{CU}}\sum_{i=1}^{N_{CU}}N_{ACim}$, where $N_{SIM}$ is the total number of frames per simulation. Then, $\delta_{AC}$ is defined as $\delta_{AC}=\frac{N_{AC}}{K}$.
- $\delta_{NAC}$ is the average ratio of the number of non-overlapping available channels to that of available channels. This parameter indicates the average percentage of non-overlapping available channels among all available channels. The average number of non-overlapping available channels of all CUs is given as $N_{NAC}=\frac{1}{N_{SIM}}\sum_{m=1}^{N_{SIM}}\frac{1}{N_{CU}}\sum_{i=1}^{N_{CU}}N_{NACim}$. Then, $\delta_{NAC}$ is defined as $\delta_{NAC}=\frac{N_{NAC}}{N_{AC}}$.
- $\delta_{OAC}$ is the average ratio of the number of overlapping available channels to that of available channels. This metric implies the average percentage of overlapping available channels across all available channels. $\delta_{OAC}$ is defined as ${1-\delta }_{NAC}$.

The simulation is executed using the MATLAB software under the following conditions:

- We consider a three-dimensional network topology (refer to Figure 1), as well as the time and frequency domain fragmentation (refer to Figure 2) as specified in Section 2.
- The values of $Z_{MAX}$ and r of Figure 1 are given as 1000 and 5000 m, respectively.
- The pair of $N_{CU}$ and K is varied as [(10,50), (25,50), (50,50), (25,25), (25,125)] to evaluate the impact of $N_{CU}$ and K on the proposed channel allocation method.
- The values of α and $T_{NS}$ are determined based on the analysis in [14] and are given as 5 and 10 s, respectively. Therefore, the length of a frame is calculated as $T=\left(1+\alpha \right)\times T_{NS}$.
- The asymmetry of the available channels of CUs is due to the spatiotemporally random occurrence of NCUs. This asymmetry can be generated by randomly setting the number, time, and duration of NCUs occurring on each channel. To do this, $T_{MAX}$ is set equal to T, implying that the occurrence time duration of an NCU is uniformly distributed in the range of [1, *T*]. The occurrence time of an NCU is also uniformly distributed in the range of [1, *T*]. From these conditions, an NCU can exist over two frames at most. In addition, the number of NCUs that occur at one channel per frame follows a Poisson distribution with an average number of NCUs per frame, $\lambda_{NCU}$, varying from 0 to 5.0 in steps of 0.5. For example, $\lambda_{NCU}=0$ implies no NCUs at a channel.
- The simulation time, expressed as the number of total frames ($N_{SIM}$), is set to $10^{6}$.

In the simulations, the following assumptions are made:

- It is assumed that the mobility of an NCU does not affect a CU’s sensing during a single frame. In other words, once an NCU is detected by a CU in one frame, its status remains unchanged during that frame.
- The location of a CU is assumed to be fixed, thus maintaining the channel allocation result during a frame. The issue of an assigned channel becoming unusable due to CU movement is related to spectrum mobility (or handoff), which falls beyond the scope of designing an efficient channel allocation method.
  To investigate the pure effect of $\lambda_{NCU},N_{CU}$, and K, it is also assumed that all sensing information is transmitted successfully without errors.

#### 4.1.2. Results

**Figure 9:**
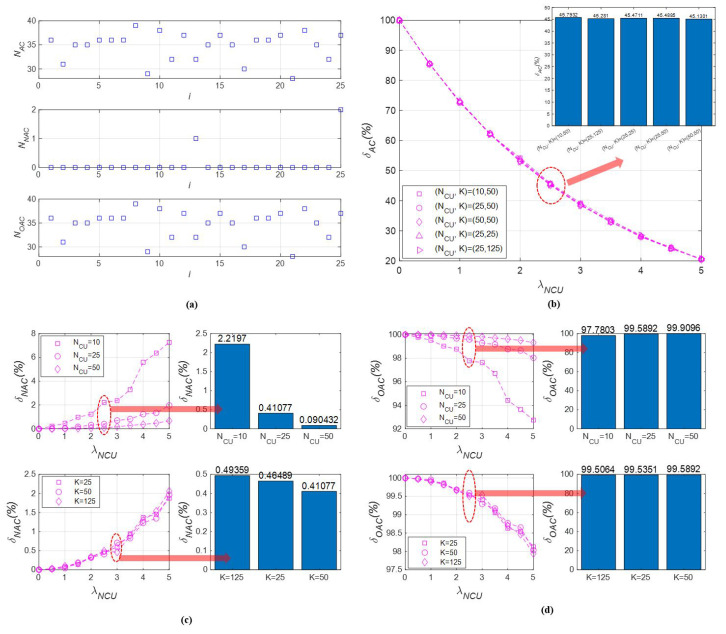
The results of the asymmetry of available channels analysis: (**a**) a snapshot of $N_{AC},N_{NAC},$ and $N_{OAC}$ at $N_{CU}$ of 25, K of 50, and $\lambda_{NCU}$ of 1.5; (**b**) $\delta_{AC}$ according to $\lambda_{NCU},K,$ and $N_{CU}$; (**c**) $\delta_{NAC}$ according to $\lambda_{NCU},K,$ and $N_{CU}$; (**d**) $\delta_{OAC}$ according to $\lambda_{NCU},K,$ and $N_{CU}$.

This section analyzes the asymmetry of available channels among CUs. To demonstrate this, Figure 9a shows the values of $N_{AC},N_{NAC},$ and $N_{OAC}$ at a particular instance with $N_{CU}$ of 25, K of 50, and $\lambda_{NCU}$ of 1.5. In this snapshot, the values of $N_{AC},N_{NAC},$ and $N_{OAC}$ are within the range of [29, 39], [0, 2], and [29, 39], respectively. It can be observed that the values of $N_{AC},N_{NAC},$ and $N_{OAC}$ for all CUs are inconsistent, without any apparent pattern. This straightforward result confirms that the characteristics of the available channels for CUs are asymmetrical.

In addition, the simulation results of $\delta_{AC}$, $\delta_{NAC}$, and $\delta_{OAC}$ are obtained by varying the average number of NCUs occurring at a channel, the number of channels, and the number of CUs. First, the results of $\delta_{AC}$ are presented as follows:

- As shown in Figure 9b, $\delta_{AC}$ is inversely proportional to $\lambda_{NCU}$. This result is intuitive, as the more NCUs that exist in the UCAN, the fewer available channels are left. When the average number of NCUs per channel is approximately 2 to 3, $\delta_{AC}$ drops to approximately 50%. This demonstrates that the presence of NCUs around a CU can significantly restrict the number of available channels.
- As shown in the bar graph in the upper right of Figure 9b, the results for $\delta_{AC}$ for five pairs of ($K,N_{CU}$) show almost similar performance. $N_{ACim}$ represents the number of available channels experienced by only CU i. When determining $N_{ACim}$, other CUs in the UCAN have no effect. As a result, $\delta_{ACim}$ for the CU is also unaffected by the presence of other CUs. Since $\delta_{AC}$ is the average of the $\delta_{ACim}$ values for all CUs, the number of CUs does not have an impact on the overall performance of $\delta_{AC}$.
- An increase in the number of channels has also little impact on $\delta_{AC}$. This is because the simulations are performed under the assumption that NCUs occur on average in all channels. This results in an insignificant change in $\delta_{AC}$, regardless of the number of channels. However, the number of available channels increases proportionally to the number of channels.
- As a result, the value of $\frac{K}{N_{CU}}$ can only be taken as an upper bound in predicting the number of channels that can be allocated to a CU before channel allocation. The number of average NCUs present at a channel, which is uncontrollable, directly affects $\delta_{AC}$. If the number of NCUs is high, only a limited number of channels can be used, regardless of the total number of channels. Ignoring this situation and using unavailable channels will result in frequent communication failures due to collisions with NCUs.

Second, the results of $\delta_{NAC}$ are summarized as follows:

- It is observed that the occurrence of non-overlapping available channels is rare among the available channels. As shown in Figure 9c, the values of $\delta_{NAC}$ range from 0 to 8%. When there are no NCUs ($\lambda_{NCU}=0$), there are no non-overlapping available channels.
- As $\lambda_{NCU}$ decreases, the number of non-overlapping available channels also decreases. When the number of NCUs decreases, more channels can be exposed to CUs. Consequently, the probability of a channel being available to only a specific CU decreases significantly.
- As $N_{CU}$ increases, $\delta_{NAC}$ decreases, as depicted in the bar graph at the upper right of Figure 9c. This is because the probability of sensing available channels increases with the number of CUs. This leads to a decrease in the number of non-overlapping available channels. However, the difference in $\delta_{NAC}$ performance due to $N_{CU}$ is negligible.
- As shown in the bar graph at the lower right of Figure 9c, the performance of $\delta_{NAC}$ also decreases as K increases. While a larger number of channels may increase the probability of non-overlapping available channels, the effect of K on $\delta_{NAC}$ is much smaller compared to the effect of $N_{CU}$ and $\lambda_{NCU}$ on $\delta_{NAC}$.
- As a result, it is confirmed that the proportion of non-overlapping available channels among available channels is low, making it difficult to assess the impact of the simulation conditions.

Third, the results of $\delta_{OAC}$ are explained as follows:

- It can be observed that the majority of available channels are shared among CUs under the given conditions. As depicted in Figure 9d, $\delta_{OAC}$ is over 92%;
- The trend of $\delta_{OAC}$ with respect to $\lambda_{NCU},K$, and $N_{CU}$ is exactly the reverse of the trend of $\delta_{NAC}$ with respect to the same parameters, since $\delta_{OAC}$ is defined as ${1-\delta }_{NAC}$.

In conclusion, most of the available channels are commonly feasible to most of the CUs, and it can be confirmed that this phenomenon becomes apparent as the number of NCUs decreases. Additionally, the number of available channels can be predicted by the upper bound of the number of CUs and the number of channels, but the number of NCUs actually affects the asymmetry of available channels in a UCAN.

### 4.2. Permance Analysis of EMRRA

#### 4.2.1. Performance Metrics and Simulation Conditions

The performance of EMRRA and MRRA is evaluated using the following four performance metrics:

- The channel allocation rate, CAR, is the average ratio of the number of allocated channels to that of the total channels. This performance metric shows the availability of channels among all channels. The CAR for CU i at the $m_{th}$ frame, ${CAR}_{im}$, is expressed as ${CAR}_{im}=\frac{N_{Oim}}{K}$. Thus, CAR is defined as $\frac{1}{N_{SIM}}\sum_{m=1}^{N_{SIM}}\frac{1}{N_{CU}}\sum_{i=1}^{N_{CU}}{CAR}_{im}$.
- Fairness, f, is the average of the fairness indices of all CUs. This performance metric indicates the difference in channel allocation rate among all CUs. The fairness index at the $m_{th}$ frame, $f_{m}$, is calculated using ${CAR}_{im}$ and the formula of Jain’s index in [34]. Thus, $f_{m}$ is expressed as $f_{m}=\frac{{\left(\sum_{i=1}^{N_{CU}}{CAR}_{im}\right)}^{2}}{N_{CU}\times \sum_{i=1}^{N_{CU}}{{CAR}_{im}}^{2}}$. Finally, f is defined as $\frac{1}{N_{SIM}}\sum_{m=1}^{N_{SIM}}f_{m}$.
- The drop rate, DR, is the average ratio of the number of dropped channels to that of channels corresponding to the volume of ingress traffic (i.e., $N_{Iim}$). This performance metric shows how much of the input traffic is dropped in terms of the number of channels. The drop rates for CU i at the $m_{th}$ frame, ${DR}_{im}$ and DR, are defined as $\frac{N_{Dim}}{N_{Iim}}$ and $\frac{1}{N_{SIM}}\sum_{m=1}^{N_{SIM}}\frac{1}{N_{CU}}\sum_{i=1}^{N_{CU}}{DR}_{im}$, respectively.
- The collision rate, CR, is the average ratio of the number of collided channels to that of allocated channels. This performance metric represents how many channels collide among the allocated channels. The collision rate for CU i at the $m_{th}$ frame, ${CR}_{im}$, is represented as ${CR}_{im}=\frac{N_{CRim}}{N_{Oim}}$. Thus, CR is defined as $\frac{1}{N_{SIM}}\sum_{m=1}^{N_{SIM}}\frac{1}{N_{CU}}\sum_{i=1}^{N_{CU}}{CR}_{im}$.

The simulation is also conducted using the MATLAB software under the following conditions:

- Topology- and time-related conditions, including $Z_{MAX}$, r, α, and $T_{NS}$, are the same as described in Section 4.1.1.
- The simulation is executed under the same assumptions as outlined in Section 4.1.1.
- The value of $\frac{K}{N_{CU}}$ indicates the upper bound of the number of channels that a CU can allocate. Thus, we fix the value of K and vary that of $N_{CU}$. The number of channels, K, is given as 50, and the number of CUs, $N_{CU}$, is varied and set to [10, 25, 50].
- $T_{MAX}$ is set to T, and $\lambda_{NCU}$ is set to [0, 2.5, 5.0].
- The maximum queue length is set to 10×K.
- As explained earlier, the volume of ingress traffic for CU i at the $m_{th}$ frame, $N_{Iim}$, is expressed as the number of channels. $N_{Iim}$ has a uniform distribution in the range of [1,$\frac{2\;\times \;K}{N_{CU}}-1$]. The range of $N_{Iim}$ is determined to make its average $\frac{K}{N_{CU}}$.
- The simulation time expressed by the number of total frames (i.e., $N_{SIM}$) is set to $10^{6}$.
- The low-CAR channel allocation priority, where a channel is allocated to CUs in ascending order of the channel allocation rate, is commonly employed in EMRRA and MRRA in order to compare the performance of the two allocation methods under the same conditions.

#### 4.2.2. Results

**Figure 10:**
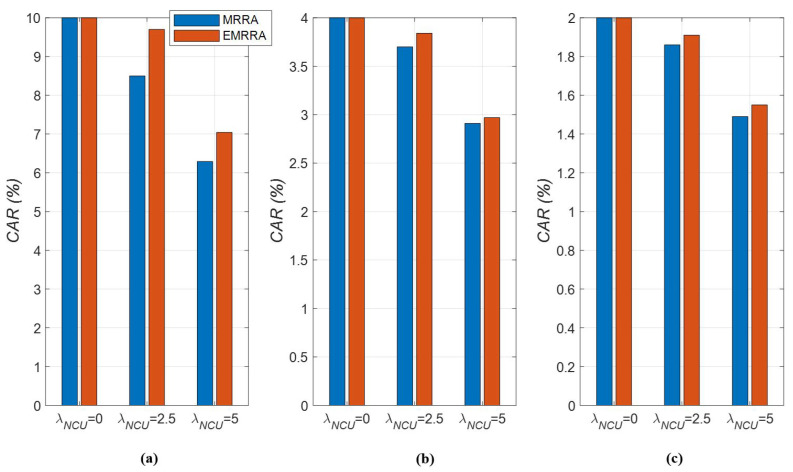
The channel allocation rates of EMRRA and MRRA with respect to $\lambda_{NCU},\;K,\;and\;N_{CU}$: (**a**) $N_{CU}=10,K=50$; (**b**) $N_{CU}=25,K=50$; (**c**) $N_{CU}=50,K=50$.

First, the results of CAR are described as follows:

- It is shown that the value of $\frac{K}{N_{CU}}$ does not have a remarkable impact on the CAR performance of the two allocation methods, as depicted in Figure 10. Instead, the increment in $\frac{K}{N_{CU}}$ improves the CAR proportionally. As the value of $\frac{K}{N_{CU}}$ increases, the upper bound of the number of allocated channels also increases. Since CAR is proportional to this upper bound, CAR also rises as well.
- As the value of $\lambda_{NCU}$ increases, the number of NCUs occurring in a channel also increases, which leads to a decrease in the number of allocated channels, as illustrated in Figure 10a–c. When the number of NCUs increases, that of the available channels that CUs can allocate decreases; this situation causes a decrease in the number of allocated channels. Therefore, it can be seen that CAR also decreases as $\lambda_{NCU}$ increases.
- We can also compare the channel allocation rates of the two channel allocation methods. In Figure 10, the red-colored bar graph shows the CAR of EMRRA; the blue-colored bar graph indicates that of MRRA. From the results, it can be seen that the performance of EMRRA is superior to that of MRRA, regardless of the values of $\lambda_{NCU}$ and $N_{CU}$, with the exception of the results at $\lambda_{NCU}=0$, as shown in Figure 10. This implies that there is no CAR difference between EMRRA and MRRA when a UCAN is interference-free. If $\lambda_{NCU}>0$, EMRRA can improve the CAR performance by 2% to 14% compared to MRRA. In particular, the difference in CAR performance between the two channel allocation methods increases as the number of $N_{CU}$ decreases (i.e., when the number of channels that one CU can be assigned increases).
- It is confirmed that the performance of CAR can be enhanced as the number of NCUs decreases or the value of $\frac{K}{N_{CU}}$ increases. Based on this result, in the area where NCUs frequently occur during channel sharing, it is recommended to set the value of $\frac{K}{N_{CU}}$ to be high.

**Figure 11:**
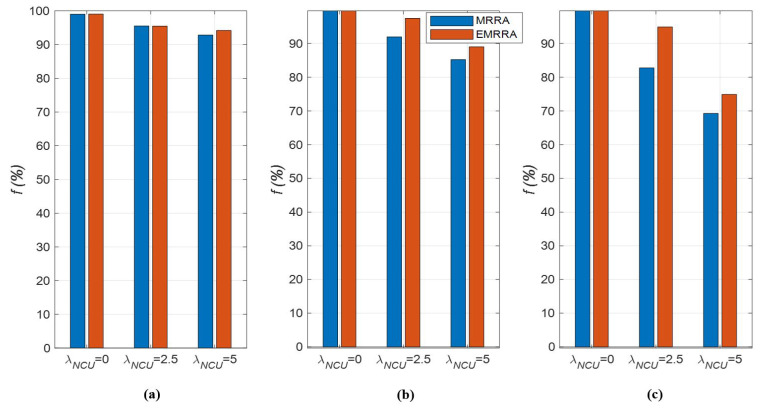
The fairness of EMRRA and MRRA with respect to $\lambda_{NCU},K,and\;N_{CU}:$ (**a**) $N_{CU}=10,K=50$; (**b**) $N_{CU}=25,K=50$; (**c**) $N_{CU}=50,K=50$.

Second, the results of fairness, f, are explained as follows:

- The fairness of the two channel allocation methods deteriorates as the value of $\lambda_{NCU}$ increases, as illustrated in Figure 11. This is caused by the fact that not only the CARs of CUs decrease as the number of NCUs increases, but also the difference in the CAR values increases due to the rise in the randomness of CAR performance.
- As the value of $\frac{K}{N_{CU}}$ decreases, the variation in fairness increases, as shown in Figure 11c. This is because as the number of allocated channels increases, the randomness of NCUs in each channel is more pronounced, leading to more severe fluctuations in the fairness results.
- Unlike the CAR performance, fairness does not always increase or decrease consistently in response to changes in simulation conditions. However, it is generally observed that fairness improves as the value of $\frac{K}{N_{CU}}$ increases and that of $\lambda_{NCU}$ decreases. In other words, when there are many allocated channels available to CUs in an environment where there are few NCUs, the fairness performance can be remarkably enhanced.
- When comparing EMRRA and MRRA in terms of fairness performance, EMRRA also outperforms MRRA, regardless of the simulation conditions. These results show that EMRRA can improve the fairness performance by up to 15% compared to MRRA, as seen in Figure 11c.

**Figure 12:**
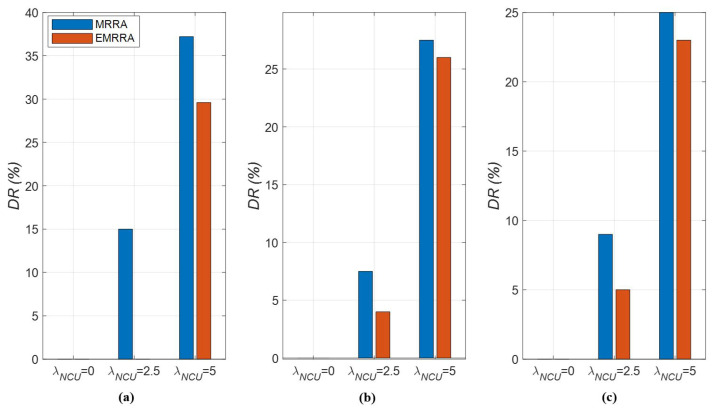
The drop rates of EMRRA and MRRA with respect to $\lambda_{NCU},K,and\;N_{CU}:$ (**a**) $N_{CU}=10,K=50$; (**b**) $N_{CU}=25,K=50$; (**c**) $N_{CU}=50,K=50$.

Third, we describe the results of the drop rate as follows:

- DR decreases as the value of $\frac{K}{N_{CU}}$ decreases, as indicated in Figure 12. As described in Section 4.2.1, the upper bound of the input traffic is given as $\frac{2\;\times \;K}{N_{CU}}-1$ to be proportional to $\frac{K}{N_{CU}}$. When the value of $\frac{K}{N_{CU}}$ decreases, the input traffic also decreases, resulting in a decrease in DR.
- On the other hand, DR increases as the value of $\lambda_{NCU}$ increases. The number of NCUs has an inverse relationship with that of the allocated channels. Thus, as the value of $\lambda_{NCU}$ increases, there are not enough allocated channels to handle the input traffic, leading to a deterioration in DR performance. Additionally, the results indicate that DR is more affected by the value of $\lambda_{NCU}$ than that of $\frac{K}{N_{CU}}$.
- When comparing EMRRA to MRRA in terms of DR, it is shown that EMRRA can reduce DR by 5% to 100% compared to MRRA, as depicted in Figure 12a–c. This performance comparison demonstrates that the improvement in DR performance is more remarkable compared to other performance metrics.

**Figure 13:**
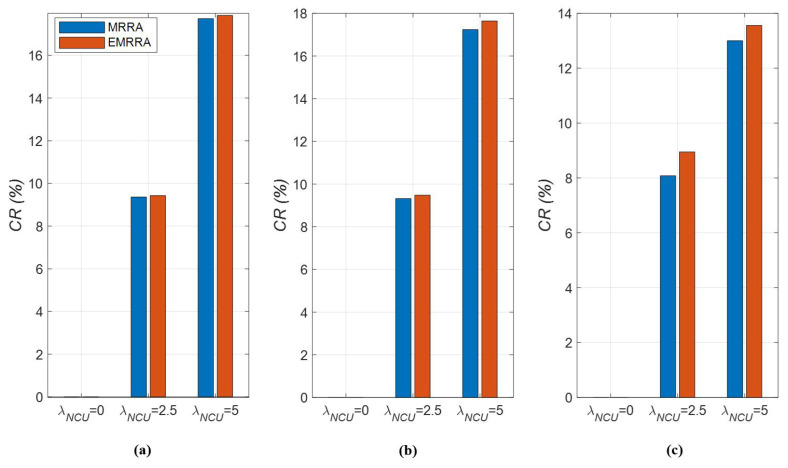
The collision rates of EMRRA and MRRA with respect to $\lambda_{NCU},K,and\;N_{CU}:$ (**a**) $N_{CU}=10,K=50$; (**b**) $N_{CU}=25,K=50$; (**c**) $N_{CU}=50;K=50$.

Fourth, the results of the collision rate are summarized as follows:

- As the value of $\frac{K}{N_{CU}}$ decreases, CR decreases, as illustrated in Figure 13. This is because a decrease in the value of $\frac{K}{N_{CU}}$ also results in a decrease in the number of allocated channels. This situation causes a reduction in the probability of collision.
- In case of $\lambda_{NCU}=0$, there are no collisions due to the absence of NCUs. As the value of $\lambda_{NCU}$ increases, CR also increases. This is a logical outcome, as NCUs cannot be distinguished from sensing and non-sensing sub-frames. In environments where many NCUs occur, multiple NCUs can exist even in the non-sensing sub-frame. In this case, the probability that a CU collides with the NCUs on its allocated channel may increase. In addition, it is shown that CR is more affected by the value of $\lambda_{NCU}$ than by that of $\frac{K}{N_{CU}}$, as depicted in Figure 13a–c.
- Unlike other performance metrics, MRRA shows slightly better performance than EMRRA in terms of CR. As the CAR results demonstrate, CUs can allocate more channels in EMRRA than in MRRA, regardless of the simulation conditions. However, an increase in the number of allocated channels also increases the probability of collision, resulting in worse CR performance for EMRRA compared to MRRA. Therefore, choosing between EMRRA and MRRA involves a trade-off between CR and other performance metrics.

## 5. Conclusions

In UCANs, the sensing results of CUs may vary due to the random occurrence of interferers. As a result, after channel sensing in one frame, the number of available channels and corresponding channel indices that a CU experiences can differ from one another. This asymmetry of the available channels may remarkably affect the efficiency and fairness of channel utilization among CUs in UCANs.

To this end, we verified the asymmetry of available channels in a centralized UCAN through simulations. Our results confirmed the existence of asymmetry under various simulation conditions, including differences in the number of available channels, non-overlapping channels, overlapping channels, and corresponding channel indices. We also found that the majority of the available channels were redundant to more than two CUs.

Then, we proposed a heuristic channel allocation method, referred to as EMRRA, to address the issue of the asymmetry of available channels in centralized UCANs. The method differs from the existing MRRA, which randomly assigns a channel to a CU in each round without considering the asymmetry. The key differences in EMRRA are as follows: (1) it selects an available channel with a low degree of redundancy when assigning a channel to a CU; (2) in the case of multiple CUs with the same allocation priority, it assigns the channel to the CU with the fewest available channels. These features of EMRRA are all intended to utilize as many channels as possible.

In addition, we conducted simulations to compare the performance of EMRRA and MRRA. The results showed that EMRRA outperformed MRRA in terms of the channel allocation rate, fairness, and drop rate but had a slightly higher collision rate. The higher collision rate of EMRRA is expected as collisions become more likely with an increase in the number of allocated channels. These results suggest that there is a trade-off between the collision rate and other performance metrics when choosing between EMRRA and MRRA.

It can be concluded that the asymmetry of the available channels is another inherent characteristic, along with long propagation delay or multi-path, in cognitive communication applied in a multi-channel UCAN. As a result, it is necessary to take the asymmetry of the available channels into account when designing protocols for UCANs, such as channel access, spectrum mobility, or channel sharing protocols.

## Figures and Tables

**Table 1:** Summary of previous works on UCANs.

Previous Work	Type	Characteristics
Wang et al. (2016) [15]	Single resource allocation	A heuristic method for spectrum decisions among cluster heads in a cluster-based underwater sensor network is proposed. In this method, a cluster head can borrow additional spectrum resources for data transmission from neighboring cluster heads by exchanging traffic information in the control channel.
Li et al. (2017) [7]		A technique for dolphin-aware data transmission in a multi-hop underwater communication network is proposed. This method optimizes the transmission schedules of CUs to maximize end-to-end throughput and reduce the harmful impact on dolphins. To do this, the technique models the stochastic nature of dolphin communications.
Baldo et al. (2008) [16]		A dynamic spectrum access scheme is proposed, which determines the CU–channel pairs to maximize the minimum channel capacity per CU using graph theory.
Luo et al. (2020) [17]		A dynamic control channel medium access control (MAC) is designed, which adaptively adjusts the bandwidth used for control by CUs based on their traffic for a distributed acoustic network.
Pottier et al. (2017) [18]		An efficient algorithm is proposed for an OFDM-based distributed underwater network that uses cognitive radio. This algorithm allocates a user’s transmission power to optimize the utility function related to the information rate using game theory.
Jin et al. (2016) [19]		A channel allocation method is designed for a distributed underwater network. This technique enables CUs to select the optimal channel to maximize the channel sharing reward.
Wu et al. (2019) [20]		A spectrum allocation technique that optimizes energy efficiency by considering the spectrum sensing errors and uncertainty of channel state information in an OFDMA-based underwater network.
Yang et al. (2015) [8]		A power control mechanism is proposed to maximize transmission power and network throughput by avoiding collisions with natural interferers using a Nash equilibrium-based utility function.
Wang et al. (2016) [21]		A QoS-driven power allocation approach is introduced for a UCAN. This method allocates a CU to an optimal power while considering statistical QoS constraints such as delay bounds.
Yun et al. (2022) [14]		A heuristic channel allocation protocol for a UCAN is proposed, which determines the efficient channel allocation priority of CUs and the channel allocation method.
Luo et al. (2017) [22]	Joint resource allocation	A resource allocation method is designed considering the traffic characteristics of neighboring sender CUs. Based on traffic conditions, the receiver CU allocates the sender CUs into a pair of channel and transmission power to maximize their transmission rate.
Li et al. (2021) [23]		A joint channel and power allocation method is designed in an OFDM-based UCAN. In this technique, the joint allocation is formulated as an optimization problem to minimize the maximum outage probability.
Le et al. (2014) [24]		An efficient spectrum management scheme is proposed. In this scheme, the receiver CU assigns the channel and power to the sender CU based on the received channel gain information, aiming to maximize the total channel capacity.
Yan et al. (2016) [25]		A joint relay selection and power allocation method in a UCAN is designed. In this method, data from CUs are forwarded by multiple relays (AUVs), considering limited feedback of quantized CSI information to achieve the maximum sum rate.
Yan et al. (2020) [26]		A joint relay selection and power allocation method is proposed in a UCAN, which takes into account a trust parameter to overcome imperfect spectrum sensing and maximize the network throughput.
Liu et al. (2019) [27]		The joint optimization of the cooperative spectrum sensing time, channel allocation, and power is studied for a UCAN in order to maximize both spectral efficiency and energy efficiency.
Tran-Dang et al. (2019) [28]	Routing	An efficient bandwidth-aware routing protocol for UCAN is proposed, which optimizes the spectrum utilization while considering the bandwidth requirements of CUs.
Chen et al. (2020) [29]		A marine mammal-friendly routing protocol is designed to improve spectrum utilization and protect underwater animals in underwater acoustic sensor networks.
Wang et al. (2017) [30]	Connectivity analysis	A study that models and analyzes the connectivity and coverage of CUs is conducted in a distributed underwater network. This approach is intended to ensure their QoS while taking into account external factors such as acoustic frequency, spreading factor, wind speed, and NCU activity.
Mishachandar (2021) [13]	Framework	A UCAN framework is introduced to enhance spectrum utilization by mitigating underwater natural and artificial interferers and by incorporating strategies for sensing, sharing, power control, interferer classification, and spectrum management.
Cheng et al. (2017) [31]		An eco-friendly framework is designed for assigning spectra by predicting interference with underwater animals through preliminary knowledge acquisition of marine mammals, channel availability prediction, channel assignment, transmission, and channel evaluation.
Yun et al. (2021) [3]	Frequency fragmentation	In this work, the standardization of the underwater acoustic frequency band using the channel raster concept of terrestrial wireless networks is performed in order to define acoustic channels for UCAN.

**Table 2:** The definition of parameters to describe the asymmetry of available channels.

Parameter	Description
$N_{CU}$	The number of CUs
i	$\mathrm{The}\;\mathrm{index}\;\mathrm{of}\;a\;\mathrm{CU}\;(1\leq i\leq N_{CU}$)
K	The number of channels
k	The index of a channel (1≤k≤K)
m	The index of a frame
$N_{NACim}$	The number of NACs for CU i$\;\mathrm{at}\;\mathrm{the}\;m_{th}$ frame
$N_{OACim}$	The number of OACs for CU i$\;\mathrm{at}\;\mathrm{the}\;m_{th}$ frame
$N_{ACim}$	The number of ACs for CU i$\;\mathrm{at}\;\mathrm{the}\;m_{th}$ $\mathrm{frame}\;(N_{ACim}=N_{NACim}+N_{OACim}$)
$M_{im}$	The set of ACs for CU i$\;\mathrm{at}\;\mathrm{the}\;m_{th}$ frame
$N_{CUkm}$	The number of CUs that can access k$\;\mathrm{at}\;\mathrm{the}\;m_{th}$ frame

## Data Availability

Not applicable.
